# On the Minim Measure

**Published:** 1817-02

**Authors:** Henry Ward


*For the London Medical and Physical Journal.*

### *On the Minim Measure;* by Mr. Henry Ward.

MR. WALKER was pleased, in the last volume of your Journal, to make a reply to a communication of mine, which was inserted in the Number for November. He there confesses the very material difference existing between the minim and the drop; at the same time aims at a method to guard the practitioner against those errors which, in this instance, is likely to take place; which, although, in that gentleman, is very laudable, yet, I am sorry to say, it is far from being correct. He says, "that two drops of any tincture or spirituous liquid, ordinarily speaking, is equal to one minim." His observation, that the minim is the sixtieth part of a drachm, is undoubtedly right. But is this sufficient to prevent those mischiefs which are likely to accrue from the use of the measure in question? And, by a reference to my Table, inserted in No. 213 of your Journal, you will find Mr. Walker's idea, respecting two drops being equal equal to one minim, totally incorrect; for in some places it considerably exceeds, and in others falls short, of the numbers there specified.

*Sloane-street; Dec.* 1816.

